# Mutations increased overexpression of *Notch*1 in T-cell acute lymphoblastic leukemia

**DOI:** 10.1186/1475-2867-12-13

**Published:** 2012-04-05

**Authors:** Chunlan Lin, Haitao Zheng, Chunyan Wang, Lijian Yang, Shaohua Chen, Bo Li, Yubing Zhou, Huo Tan, Yangqiu Li

**Affiliations:** 1 Department of biochemistry, Medical College, Jinan University, Guangzhou 510632, China; 2 Institute of Hematology, Medical College, Jinan University, Guangzhou 510632, People's Republic of China; 3 Centre of Oncology and Hematology, the First Affiliated Hospital of Guangzhou Medical College, Guangzhou 510230, China; 4 Key Laboratory for Regenerative Medicine of Ministry of Education, Jinan University, Guangzhou 510632, People's Republic of China

## Abstract

**Background:**

The *Notch *signaling pathway is crucial in T-cell development, *Notch*1 mutations are frequently present in T-cell acute lymphoblastic leukemia (T-ALL). To investigate the feature of *Notch*1 mutation and its corresponding expression level in Chinese patients with T-ALL, detection of mutation and the expression level of *Notch*1 gene was preformed using RT-PCR, sequencing and real-time PCR respectively.

**Results:**

Two *Notch*1 point mutations (V1578E and L1593P) located on HD-N domain were identified in three cases out of 13 T-ALL patients. The mutation on 4733 position (V1578E) found in two cases was a novel mutation. The overexpression of *Notch*1 was detected in all samples with T-ALL, moreover, significantly higher expression of *Notch*1 was detected in the T-ALL with *Notch*1 mutation group compared with T-ALL with WT *Notch*1 group (*p *= 0.0192).

**Conclusions:**

Higher expression of *Notch*1 was associated with *Notch*1 mutation, more novel mutation of this gene might be identified in different populations and its contribution to the molecular pathogenesis of T-ALL is needed further research.

## Background

T-cell acute lymphoblastic leukemia (T-ALL) which occurs mainly via the proliferation of malignant T cell clones, accounts for 15% of newly diagnosed ALL cases in children and 20-25% of ALL cases in adults [1]. Overall, these are aggressive malignancies that do not respond well to chemotherapy and have a poorer prognosis than their B-cell counterparts [2]. Complex acquired genetic aberrations include chromosomal translocations (frequently involving TCR), as well as gene rearrangements and mutations resulting in abnormal expression of oncogenes like *Notch*1 may be associated with the advance and resistance to treatment of this disease [3].

*Notch*1 was discovered in 1991 through analysis of rare T-cell lymphoblastic leukemia/lymphoma with balanced (7;9) translocation [4]. Acquired *Notch*1 mutations are present in about 50% of T-ALL [5,6]. More than hundred different mutations frequently involved in heterodimerization domain (HD), transactivation domain (TAD) and praline, glutamic acid, serine, threonine-rich (PEST) domains of *Notch*1 were reported in patients with T-ALL from a lot of researcher groups in different countries [5-10]. Little is known the incidence and feature of *Notch*1 mutations in Chinese T-ALL patients [10], in this study, we detected the *Notch*1 mutations in 13 Chinese patients with T-ALL and analyzed the corresponding expression level of *Notch*1 gene.

## Materials and methods

### Samples

Thirteen newly diagnosed and untreated cases of T-ALL, 11 males and 2 females (6-55 years old; median age: 23.5 years) were included in this study, along with 20 healthy individuals as controls. The samples were collected with informed consent. All procedures were conducted in accordance with the guidelines of the medical ethics committees of the Health Bureau of Guangdong Province, China. The peripheral blood mononuclear cells (PBMCs), RNA extraction using Trizol reagent (Trizol^®^, Invitrogen, Carlsbad, CA, USA) and cDNA synthesis using random hexamer primers and reverse transcriptase (SuperScript^® ^III, Invitrogen, Carlsbad, CA, USA) were performed according to the manufacturer's instructions.

### RT-PCR and sequencing

To amplify different domain and exon of *Notch*1 according the structure of the *Notch*1 gene, 4-pair primers were purchased, which covered different exons, where the mutations happen frequently (Table 1) [5,6]. RT-PCR was performed as our previous study, positive control (Jurkat cell line) and negative control (non-template) were included in each reaction [11,12]. The PCR products were directly sequenced using a BigDye Terminator v3.1 Cycle Sequencing kit (Perkin Elmer, ABI) and the ABI PRISM 3100-Avant genetic analyzer. The sequences from different samples of T-ALL were analyzed with the BLAST software (http://blast.ncbi.nlm.nih.gov/Blast.cgi) to identify the mutations of *Notch*1 gene.

**Table 1:** List of the primers used in RT-PCR and real-time PCR for *Notch*1 gene amplification

Primer ^5,6^	Sequence	Domain	PCR	Function
notch26 + 27-F	5'-ACGACCAGTACTGCAAGGACC - 3'	HD/exon 26 + 27	RT-PCR	Sense
			
notch26 + 27-R	5'- AAGAACAGAAGCACAAAGGCG - 3'			Antisense

notch28-F	5'- TCGCTGGGCAGCCTCAACATCC - 3'	HD/exon28	RT-PCR	Sense
			
notch28-R	5'- ACTCATTCTGGTTGTCGTCC - 3'			Antisense

Notch TAD-F	5'- GCCCTCCCCGTTCCAGCAGTCT - 3'	TAD	RT-PCR	Sense
		
Notch TAD-R	5'- GCCTGGCTCGGCTCTCCACTCA - 3'			Antisense

Notch PEST-F	5'- CAGATGCAGCAGCAGAACCTG - 3'	PEST/exon34	RT-PCR	Sense
			
Notch PEST-R	5'- AAAGGAAGCCGGGGTCTCGT - 3'			Antisense

Notch1-f	5'-GCGACAACGCCTACCTCT-3'		real-time PCR	Sense
			
Notch1-r	5'-CTGCTGGCACAGTCATCC-3'			Antisense

β2M-f	5'-TACACTGAATTCACCCCCAC-3'		real-time PCR	Sense
			
β2M-r	5'-CATCCAATCCAAATGCGGCA-3'			Antisense

Note: *HD *heterodimerization domain; *TAD *transactivation domain; *PEST *PEST domain.

### Real-time quantitative RT-PCR (qRT-PCR)

Expression levels of *Notch1 *and the reference gene *β2M *were determined by SYBR Green I real-time PCR. PCR was performed as our previous description [12,13]. The 2^(-ΔΔT) ^method was used to present the data of the genes of interest relative to an internal control gene [12-14]. The sequences of primers used in qRT-PCR were listed in Table 1.

### Statistical analysis

Univariate analyses were done using the Mann-Whitney test to compare manes of *Notch1 *expression level between T-ALL with *Notch*1 mutations or with wild-type (WT) *Notch*1 status. *P *< 0.05 was considered as statistically significant.

## Results

### *Notch*1 mutation and polymorphirsm in T-ALL

**Figure 1:**
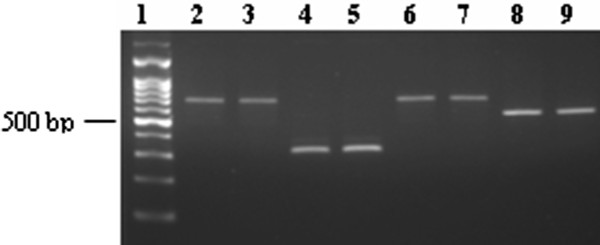
**Results of RT-PCR amplification for *Notch1 *gene using different primer pairs which located in different domains**. 1: 100 bp DNA ladder, 2-3: Amplicon of Exon 26 and 27 (652 bp), 4-5: Amplicon of Exon 28 (316 bp), 6-7: Amplicon of TAD domain (666 bp), 8-9: Ampilcon of PEST domain (530 bp).

**Figure 2:**
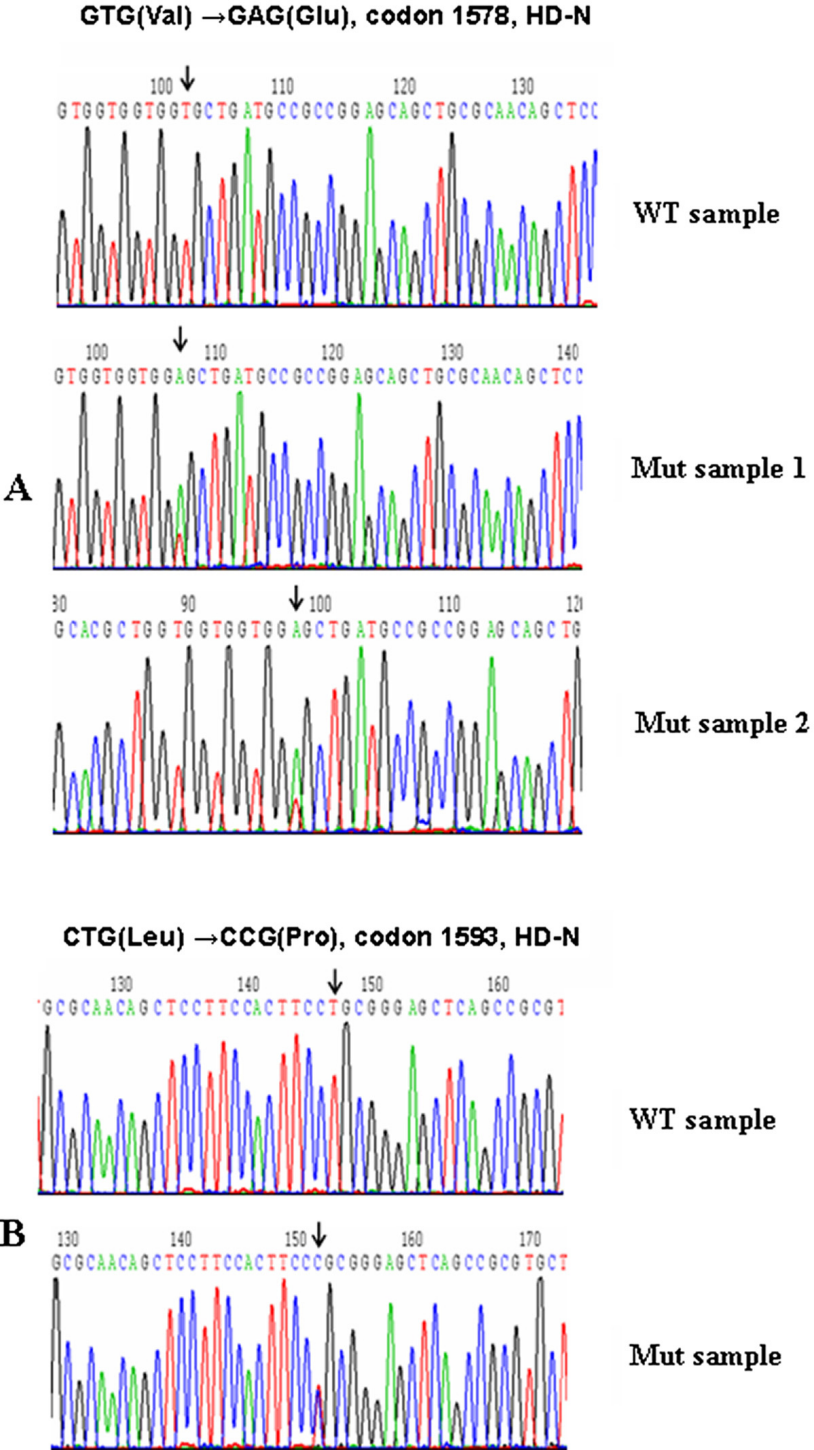
***Notch*1 mutations in 3 cases with T-ALL (Mut sample) compared with a normal sequence of *Notch*1 (WT sample)**. Heterozygous mutations were identified in position 4733 (codon 1578) and 4778 (codon 1593) in *Notch*1 gene.

*Notch*1 expression was detected in all of 13 cases with T-ALL by RT-PCR (Figure 1). The search for *Notch*1 mutation was performed in exon 26, 27 and 28 (HD), TAD and PEST domain. The PCR products were direct sequenced and revealed two mutations at nucleotide position 4733 and 4778 respectively. The mutation on 4733 position was identified in two different cases, the nucleotide sequence was changed from GTG to GAG (codon 1578), thereby leading to an amino acid exchange (Valine to Glutamic), this was a novel mutation. While the mutation on 4778 position was identified in one case, the nucleotide sequence was changed from CTG to CCG (codon 1593), leading to an amino acid exchange from Leucine to Proline. All of mutations presented were heterozygous genetic alteration. And the mutations were located on HD N-terminus (Figure 2). Sequence analysis of *Notch*1 segments revealed further a nucleotide exchange in all samples analyzed at position 5094, where we found a cytosine instead of a thymine, the alteration affected the third position of the codon 1698 and did not alter the amino acid sequence (data not shown).

### Expression level of *Notch*1 in T-ALL

**Figure 3:**
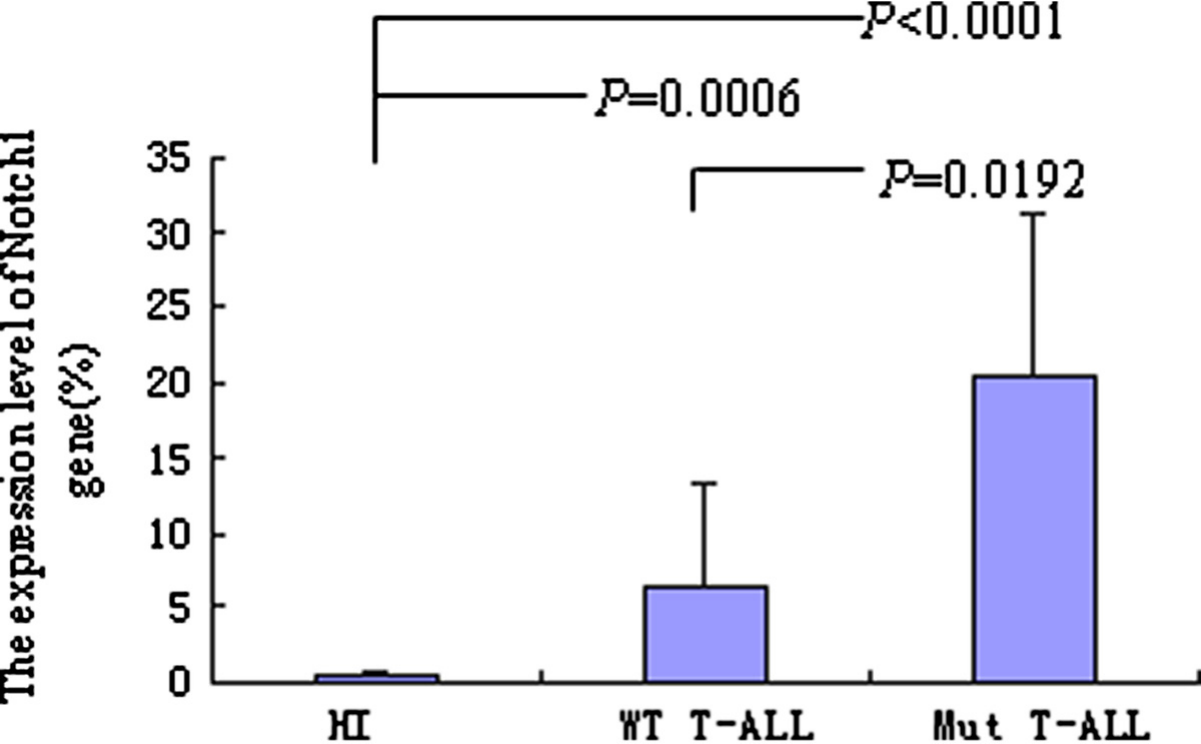
**The expression levels of *Notch*1 gene in T-ALL with *Notch*1 mutation (Mut T-ALL), T-ALL with wild type *Notch*1 (WT T-ALL) and healthy individuals (HI) groups**.

In order to compare the expression level of *Notch*1 gene in different T-ALL samples with *Notch*1 mutations or with wild-type (WT) *Notch*1 status, the expression level of *Notch*1 gene was analyzed by real-time PCR, in comparison with healthy controls (0.37 ± 0.33), significantly higher expression of *Notch1 *was found in both T-ALL with WT *Notch*1 (6.34 ± 7.03) (*p *= 0.0006) or with *Notch*1 mutations (Mut *Notch*1) (20.47 ± 10.81) (*p *< 0.0001) (Figure 3). Moreover, significantly higher expression of *Notch*1 was detected in the T-ALL with Mut *Notch*1 group compared with WT *Notch*1 group (*p *= 0.0192) (Figure 3). Interesting, the expression level of *Notch1 *in two cases with WT *Notch*1 T-ALL (24.28 and 9.06 respectively), which were not identified any mutation in the present study, was as high as the samples with *Notch*1 mutation.

## Discussion

*Notch*1 signaling is crucial for T-cell differentiation and proliferation, mutational activation of *Notch*1 is an important factor in T-ALL pathogenesis [5,7]. Translocation and mutations of *Notch*1 may alter its function resulting in overexpression and independent activation [15]. In this study, *Notch*1 mutations were identified in 3 Chinese patients with T-ALL, the incidence of *Notch*1 mutation was only 23.08% (3/13), it seemed relatively low in comparison with previous studies from different European and American countries [5,6,16]. There are rare studies described the incidence of *Notch *1 mutation in Chinese cases, one report by Zhu *et al *showed that *Notch*1 mutation was found in 29 patient out of 77 cases (37.7%) with Chinese T-ALL [10]. Similar incidence (22%) of *Notch *1 mutations was reported by a research group from Turkey [8]. However, further research is needed to collect and investigate more samples and find out the representational results.

The higher frequency of *Notch*1 mutation is found in HD, TAD and PEST domains [5-10]. In the present study, we used the 4 pair primers covered all the HD, TAD and PEST domains to amplify and sequence. Two mutations were identified in three cases, one was the mutation on 4778 position (L1593P) which was reported in previous studies [10,17], while the another mutation was identified on 4733 position (V1578E) in two different cases with T-ALL, to our best knowledge, this is a novel identified mutation. The effect of the novel mutation is needed to evaluate by further functional analysis. Both mutations were located at HD N-terminus (HD-N) domain. The *Notch*1 HD-N mutation may destabilize the subunit interaction and do not require the ligand-binding domain to activate signaling, resulting in constitutive *Notch*1 activation and subsequent cell transformation [9,17]. Based on the results, we compared the expression level of *Notch*1 in T-ALL with WT *Notch*1 or with Mut *Notch*1 group, definitive result indicated that the expression level of *Notch*1 was significant associated with *Notch*1 mutation in HD-N domain, significantly higher expression of *Notch*1 was detected in the T-ALL with Mut *Notch*1 group compared with WT *Notch*1 T-ALL group. However, overexpression of *Notch*1 was a common feature in all T-ALL patients, whether the mechanism of *Notch*1 overexpression in mutation or WT samples is different, it remains an open question. Although no mutation was detected in HD, TAD or PEST domains, high expression level of Notch*1 *in two cases with T-ALL in the present study was thought that might has potential mutation in the other domains, whole *notch*1 gene sequence analysis for these cases is needed to follow up.

In the present study, we were unable to identify mutation of *Notch*1 in PEST domain which regulates protein turnover by targeting proteins to the ubiquitin-proteosome complex for subsequent degradation [9,15]. However, a high incidence (4/15 cases) of *Notch*1 mutation in PEST domain was reported in Indian T-ALL patients [9], while lower incidence was described from a study in Chinese T-ALL patients (5/77, 6.5%) [15], as well as in Turkish patients (7%) and German patients (8.2%) [7,18], the difference may due to the racial diversify.

In summary, *Notch*1 mutations including a novel mutation were identified in a small cohort of Chinese T-ALL cases, and concomitant significantly higher expression level of *Notch*1 was found. More ongoing study was performed to follow up its predictive value and to elucidate its contribution to the molecular pathogenesis of T-ALL.

## Competing interests

The authors declare that they have no competing interests.

## Authors' contributions

YQL contributed to concept development and study design. CLL and SHC performed PCR and sequencing, HTZ, LJY and YBZ performed the real-time PCR. CYW, LJY, BL and HT were responsible for collection of clinical data. YQL and CLL and HTZ coordinated the study and helped drafting the manuscript. All authors read and approved the final manuscript.
